# Vapor phase-grown TiO_2_ and ZnO nanoparticles inside electrospun polymer fibers and their calcination-induced organization

**DOI:** 10.1007/s00706-023-03093-0

**Published:** 2023-07-12

**Authors:** Hasan Razouq, Thomas Berger, Nicola Hüsing, Oliver Diwald

**Affiliations:** Department of Chemistry and Physics of Materials, Paris Lodron Universität Salzburg, Jakob-Haringer Str. 2a, 5020 Salzburg, Austria

**Keywords:** Electron microscopy, Aggregation, Nanostructures, X-ray structure determination, Particle coagulation, Hybrid materials

## Abstract

The spatial organization of metal oxide nanoparticles represents an important factor in the chemical utilization of resulting structures. For the production of networks that are composed of metal oxide nanoparticle chains, we dispersed vapor phase-grown TiO_2_ and ZnO nanoparticles homogeneously in an aqueous polyvinyl alcohol solution. After electrospinning, we analyzed the sizes and diameters of the compositionally homogeneous electrospun fibers and discussed the size distribution and morphology of the nanoparticles inside. Calcination-induced polymer removal gives rise to self-supported nanoparticle-based nanofibers. Particle coarsening by a factor of ~ 2 for TiO_2_ and ~ 3 for ZnO nanoparticles is observed.

**Graphical abstract:**

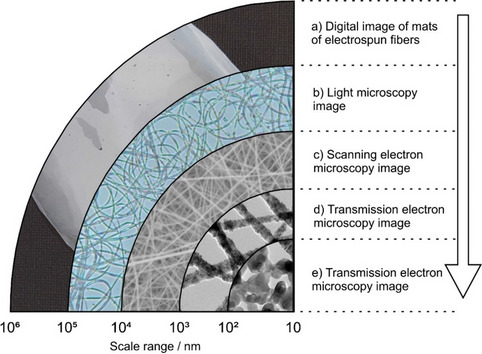

## Introduction

Structured metal oxide nanoparticle/polymer blends received continuously increasing interest in the science and technology of functional materials [1]. Relevant application fields are sensing [2–5], the design of scaffolds and membranes for regenerative biology and medicine [6, 7] or materials development for energy storage and conversion [8–11]. Here we used electrospinning to organize metal oxide nanoparticles inside the confined volume of larger-scale polymer fiber mats [12]. We used polyvinyl alcohol (PVA) as the polymer matrix. PVA is highly hydrophilic, non-toxic, biocompatible, and commonly used as an additive to prepare nanocomposites [13–16]. In addition, we formulated gas-phase synthesized TiO_2_ and ZnO nanoparticle systems that were previously established as model systems for fundamental materials research in surface science, photocatalysis, and the exploration of processing-induced property changes [1, 17, 18]. Morphological and electronic properties of TiO_2_ and ZnO nanoparticles are exploited in optoelectronic applications, light-emitting diodes, varistors, and sensing [19–22]. The properties of the oxide nanoparticle/polymer interface strongly influence the properties of the resulting composite material. This interface emerges during the different processing steps (i.e., nanoparticle formulation and electrospinning), contributing to the overall synthesis process. Importantly, nanoparticle systems of different synthetic origin are expected to interact with organic molecules differently throughout single processing steps [1, 23–26]. Moreover, many essential properties arise from the surface of the metal oxide nanoparticles [1, 17, 18, 27]. In this context, electrospinning of vapor phase-grown TiO_2_ and ZnO nanoparticle systems may allow us to generate nanoparticle-based fibers with a high surface area with retention of some properties, like photochemical activity [28], photoluminescence [18], visible light photoactivity [29], and UV-induced local heating effects [30]. Numerous studies have been reported on the electrospinning of polymer solutions containing sol–gel precursors [31, 32]. Subsequently, the electrospun metal hydroxide/polymer composite nanofibers can be converted into metal oxide nanoparticle fibers by an appropriate thermal treatment, which assures the conversion of the hydroxide into the oxide as well as the decomposition of the polymer components. In addition, there exist studies on the incorporation of preformed nanoparticles resulting from sol–gel synthesis in electrospun fibers [33–35]. However, systematic studies on incorporating vapor phase-grown metal oxide nanoparticles inside electrospun fibers are scarce [36]. Although some literature reports discuss the aggregation and organization behavior of incorporated nanoparticles into polymer solution [37, 38], the incorporation of vapor phase-grown nanoparticles has not been addressed. Here, we study the impact of colloidal processing and electrospinning on the morphological properties (primary particle size and morphology, aggregation state, microstructure) of TiO_2_ and ZnO nanoparticle ensembles prepared by vapor phase synthesis. Since the present formulation route is well suited for the exploitation of metal oxide nanoparticles into large-scale industrial applications [39], we believe that these preliminary structure characterization data represent important information for the chemical design of nanoparticle formulation.

## Results and discussion

**Fig. 1:**
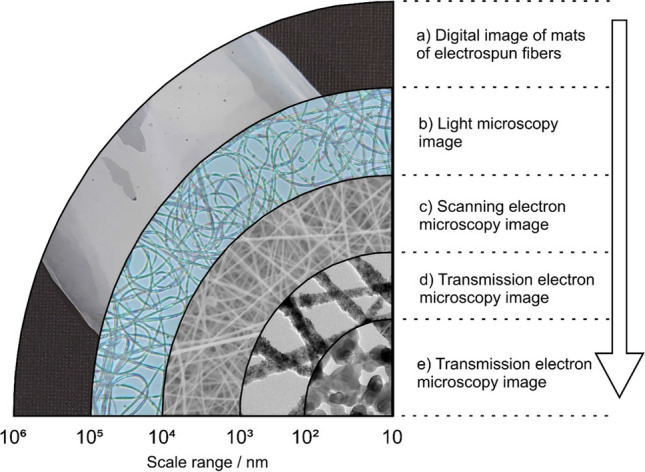
Electrospun fibers containing metal oxide nanoparticles inside the fiber volume characterized at different length scales

Figure 1 shows representative images of electrospun polymer fibers with metal oxide nanoparticles incorporated inside. The macroscale shape of the fiber arrangement is illustrated by the digital image in Fig. 1a. At microscopic observation levels of higher magnification, such sample's micro- and nanoscale structures are displayed in Fig. 1c–e, respectively.

**Fig. 2:**
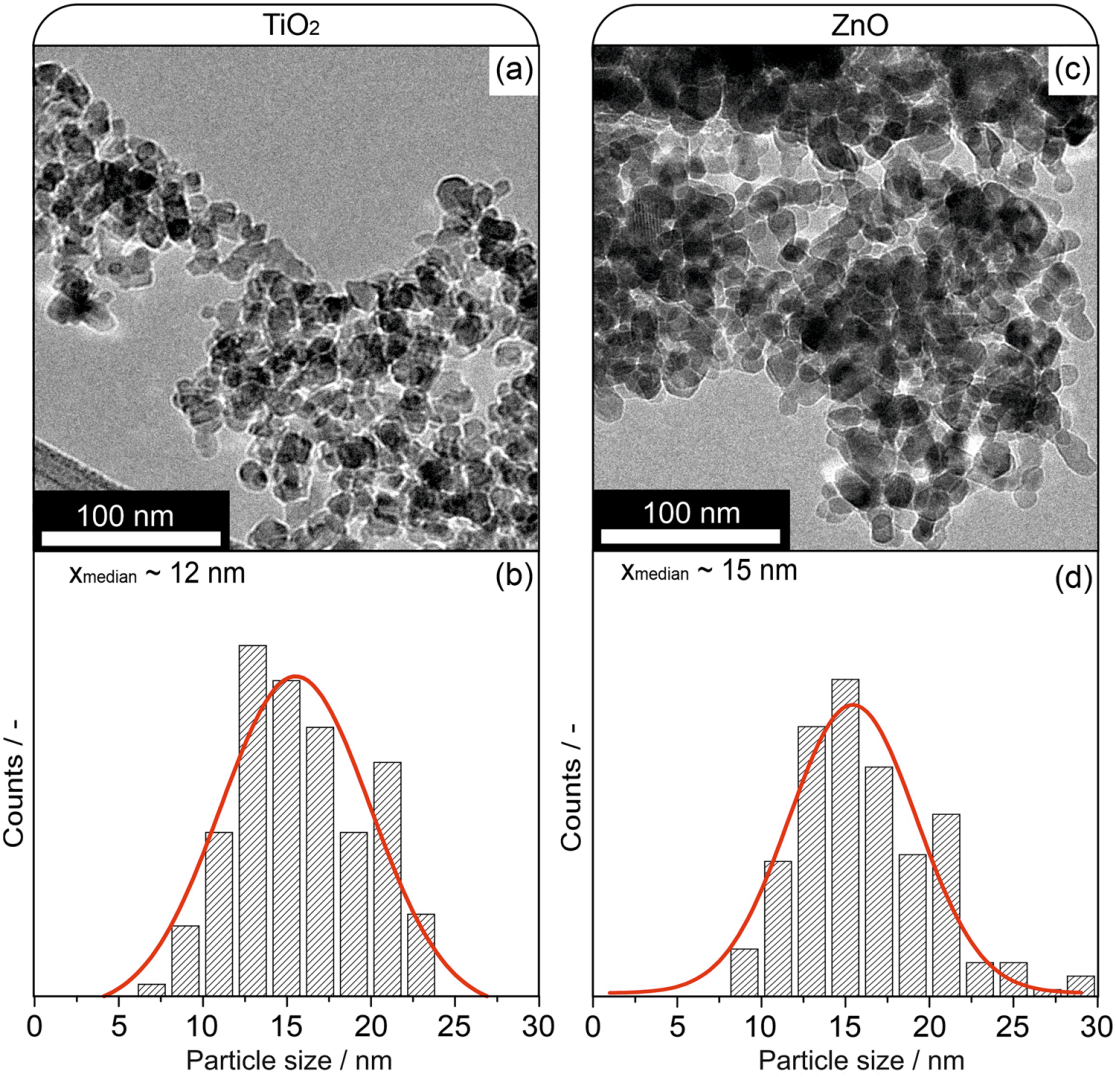
Transmission electron microscopy images of gas phase-grown annealed (**a**) TiO_2_ and (**c**) ZnO nanoparticles. The corresponding PSD plots are shown in (**b**) and (**d**) for TiO_2_ and ZnO nanoparticles, respectively

Thermally annealed gas phase-grown TiO_2_ and ZnO nanoparticles with narrow particle size distribution comprise the powders used in this study. Figure 2 shows Transmission Electron Microscopy (TEM) images and particle size distribution plots of such nanoparticles.

The TEM images of both systems show that the particles do not exhibit faceting and crystal-related habits. Moreover, the PSD plots confirm a narrow particle size distribution function with an average size of 12 nm and 15 nm for the annealed TiO_2_ and ZnO particles, respectively.

**Fig. 3:**
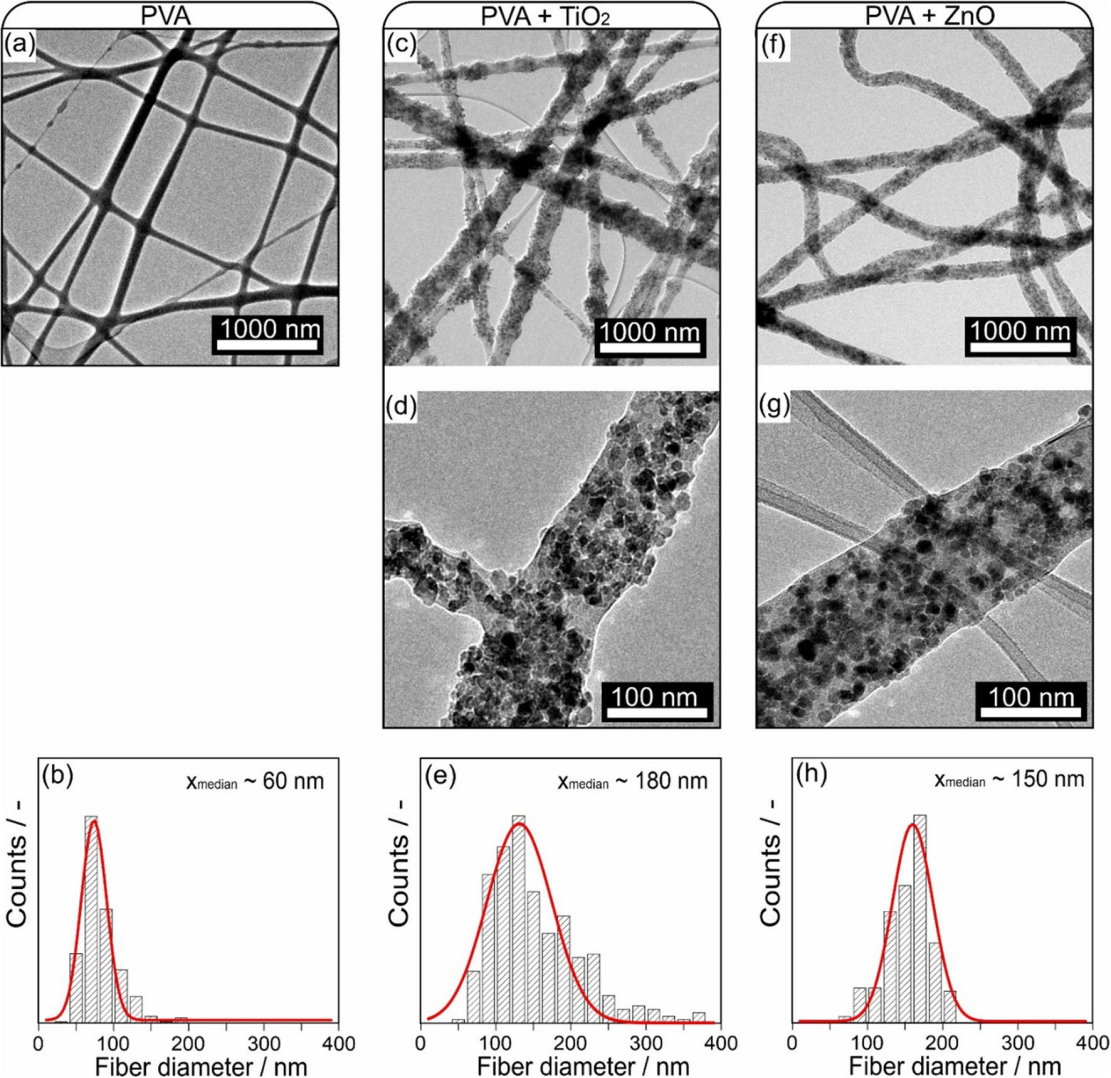
Transmission electron microscopy images with fiber diameter distribution plot of the as-spun pure polymer fibers (**a**, **b**), as-spun PVA/TiO_2_ nanoparticle fibers (**c**, **d**, and **e**), and as-spun PVA/ZnO nanoparticle fibers (**f**, **g**, and **h**). Diameter distribution plots are generated by measuring the diameters of more than 100 electrospun fibers for each sample

Table 1 lists the optimized formulation and electrospinning parameters for preparing spinnable nanoparticle–polymer mixtures and fibers derived therefrom. These parameters provide the basis for a stable and in terms of fiber homogeneity reproducible production process. Figure 3 shows transmission electron microscopy images of the as-spun fibers and the fiber diameter distribution plots.

**Table 1:** Electrospinning precursor and electrospinning process parameters for ZnO/PVA and TiO_2_/PVA nanoparticle systems

Electrospinning precursor		Electrospinning process	
Nanoparticle concentration/mg/cm^3^	60	Applied potential / kV	18
Stirring time/min	10	Distance needle tip to collector/cm	20
Sonication time/min	15	Pumping rate/cm^3^/h	0.05

The TEM images confirm the formation of homogeneous fibers from pure polymer and polymer/nanoparticle precursors (Fig. 3a, c, f). Electrospinning does not produce any changes in the size or morphology of the primary particles for both nanoparticle systems (Fig. 3d, g). The diameter of the pure polymer fibers shows a relatively narrow distribution with an average value of around 60 nm (Fig. 3b). We observed growth in the fiber diameters for the polymer nanoparticle mixtures, although identical electrospinning parameters were used. With an average final diameter value of 180 nm this growth is slightly more pronounced in the case of fibers with TiO_2_ admixture compared to the ZnO nanoparticle-loaded fiber samples with an average diameter of 150 nm. Although the TiO_2_ and ZnO PSDs are comparable, the diameter distribution of the TiO_2_ nanoparticle-loaded fibers in (e) is broader. From the statistical analysis of the microscopy data, we can conclude that loading the polymer solution with nanoparticles leads to increased width of the electrospun fibers. Unresolved differences in the aggregation behavior between TiO_2_ and ZnO nanoparticles and inside the polymer solution may explain the differences in the diameter distribution (Fig. 3e, h).

**Fig. 4:**
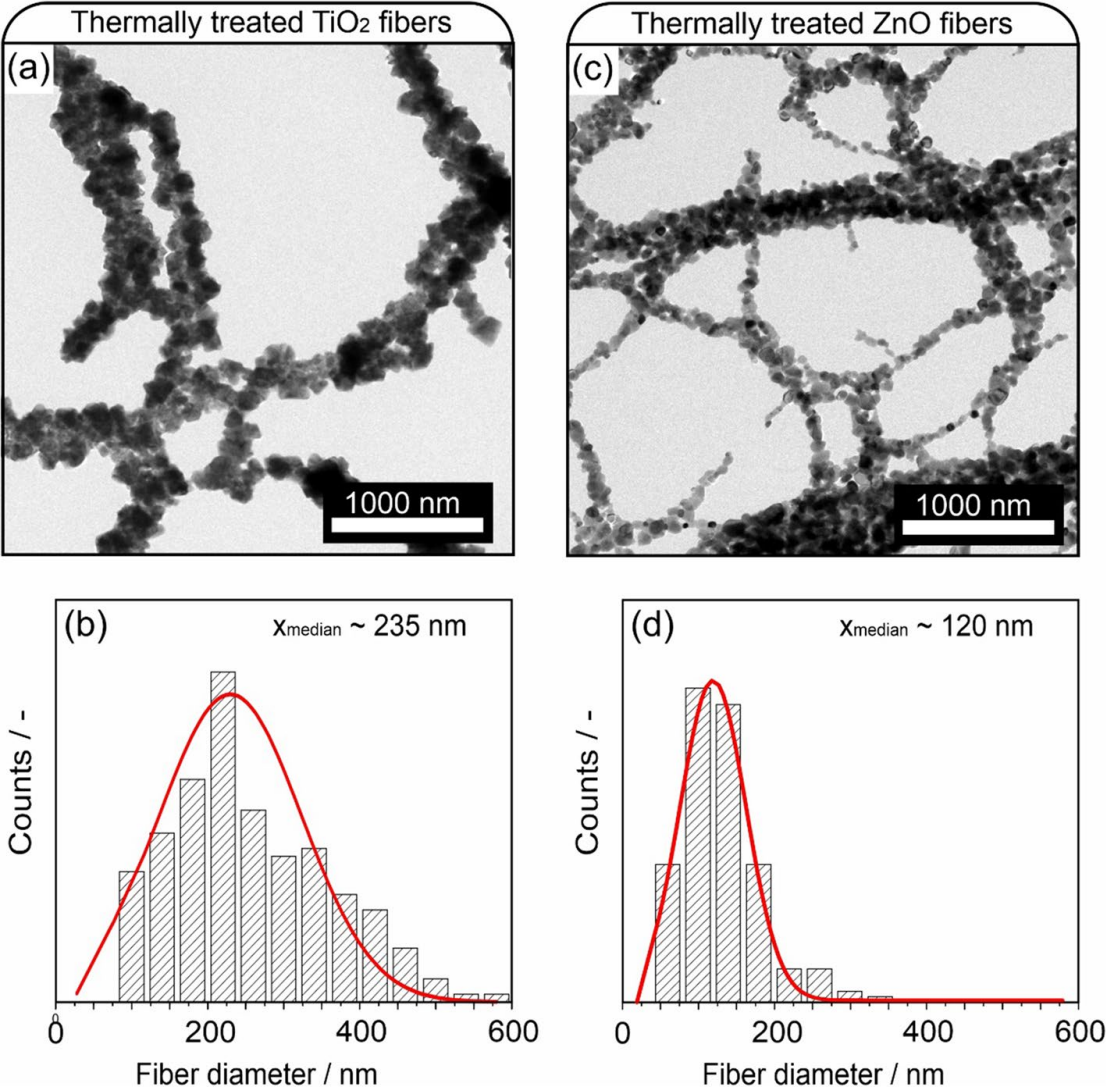
Representative TEM image of TiO_2_ nanoparticle fibers after calcination-induced polymer removal (**a**) and the corresponding fiber diameter distribution plot (**b**). TEM image of ZnO nanoparticle fibers after calcination-induced polymer removal (**c**), with fiber diameter distribution plot (**d**)

Thermal treatment up to 750 °C (with a heating rate of 5 °C min^−1^) of the as-spun fibers was performed in synthetic air to remove the polymer. Figure 4 shows TEM images of the resulting fibrous residues and corresponding fiber diameter distribution plots.

**Fig. 5:**
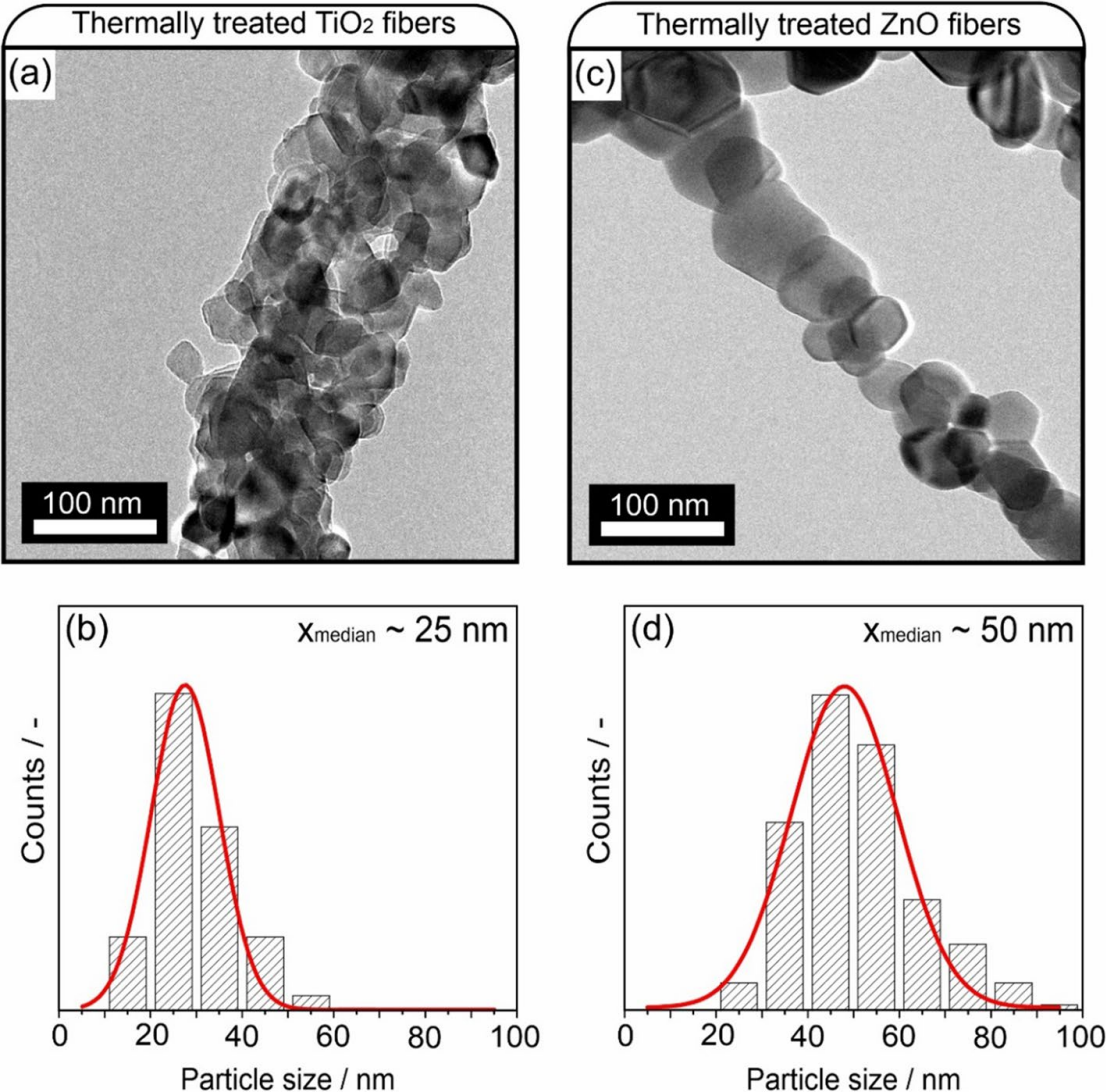
Transmission electron microscopy images of thermally treated TiO_2_ nanoparticle/polymer fibers (**a**) with the corresponding particle size distribution plot (**b**) and of thermally treated ZnO nanoparticle/polymer fibers (**c**), with particle size distribution plot (**d**)

TEM investigations reveal that non-aggregated individual nanocrystals form overlapping chains, creating a self-supported nanoparticle-based network. The fiber diameter distribution plots show that the distribution is broadened. Whereas most fibers remain in the range of diameter values specific to the as-spun fibers (Fig. 4), some fibers grow in diameter up to 600 nm. This disproportional growth is attributed to the coalescence of two or more as-spun fibers, which join during the thermal treatment to form one nanoparticle chain. Although after thermal treatment the diameters of most fibers are not changed, the size distribution of the nanoparticles inside the polymer fibers after polymer removal and formation of nanoparticle-based chains changes. A closer look at these fibers with TEM shows the individual particles that form the particle chains (Fig. 5).

The TEM images reveal sinter necks and grain boundaries between the nanoparticles to form the fibers. The size of these particles has grown as compared to those prior to thermal treatment. Whereas the average size of the TiO_2_ nanoparticles has shifted from 12 to 25 nm (factor ~ 2), the average size of ZnO nanoparticles has increased from 15 to 50 nm (factor ~ 3) after the thermal treatment.

This annealing-induced growth in particle size is inherently associated with changes in the respective specific surface areas. The repeatedly measured values for anatase TiO_2_ nanoparticle powders correspond to 110 m^2^ g^−1^ which is consistent with an average particle size of 12 nm [40]. Our estimate for the annealing-induced decrease in specific surface area – since the sample amounts obtained are too low for sorption measurements and the relative amount of grain boundary area is impossible to determine – is based on the spherically shaped TiO_2_ particles with an average particle size of 25 nm (Fig. 5b) and amounts to *A* ≤ 57 m^2^ g^−1^.

For the ZnO nanoparticles with an initial specific surface area value of 63 m^2^ g^−1^ [41] particle coarsening decreases the surface area to values below 21 m^2^ g^−1^ of the resulting structures having an average particle size of 50 nm.

The more pronounced coarsening of the embedded ZnO nanoparticles that form chains of individual sintered particles (Fig. 5c) compared to the multiple chains of smaller TiO_2_ nanoparticles (Fig. 5a) can be attributed to a combination of factors. These include chemical stability, crystal structure differences, and defect formation energies for vacancies and interstitials inside the lattice of the two metal oxides. The latter factor determines the concentration of point defects during synthesis and the ion diffusivities at the respective temperature of annealing. Minimization of surface energies related to the different faces of the two metal oxides acts as a driving force for coarsening, and the transient emergence of polar surfaces, which only exist for the ZnO wurtzite lattice, may promote particle attachment and coalescence.

Previous work revealed the disproportional growth of vapor-grown ZnO nanoparticles, where one fraction of the particles grows to larger crystals (*d* ≥ 50 nm) and another fraction retains the initial nanoparticle sizes (*d* < 20 nm) upon heating to 600 °C in high vacuum (*p* < 10^–5^ mbar) [28]. In the present case, the more uniform distribution of the ZnO particles results from the surrounding polymer matrix and the organic byproducts, which can be attributed to the thermally induced polymer decomposition in air. Related interface layers and species are expected to enhance ion mobility and mass transfer.

Growing nanoparticles inside the polymer matrix is a simple method to prepare composites and generate nanoparticle-based anisotropic structures [33, 42]. However, this method is reproducible only at a limited scale, and the final material purity could be affected by organic remnants. On the other hand, dispersing pre-synthesized nanoparticles into a polymer solution for composite formation is more efficient at larger scales [40]. The main challenge with this method is the nanoparticle stability against aggregation [38], which we overcome by determining the colloidal dispersion parameters, like the choice of polymer, solvent, nanoparticle concentration, and stirring/sonication time, in addition to the electrospinning parameters, like applied potential, pumping rate, and distance between the tip of the needle and the collector.

## Conclusion

We prepared spinnable mixtures of polymer solution and gas phase-grown nanoparticles to produce the homogeneous and beads-free as-spun fibers with narrow fiber diameter distribution. Moreover, this approach can obtain well-distributed TiO_2_ and ZnO nanoparticles with retained narrow particle size distributions in the polymer matrix. Finally, we adjusted the thermal treatment in dry air atmosphere to remove the organic polymer and obtain self-supported nanoparticle-based fibers. ZnO and TiO_2_ nanofiber networks attract attention due to their porous structures, chemical stability, non-toxicity, large specific surface area, and the opportunity for scale-up. Moreover, using well-characterized vapor phase-grown TiO_2_ and ZnO nanoparticles increases the control over the properties of the nanofiber networks. Therefore, desired physico–chemical interactions can be optimized, leading to a more comprehensive range of applications, such as sensing, nanoelectronics, optical devices, and catalysts.

## Experimental

### Powder synthesis

**Fig. 6:**
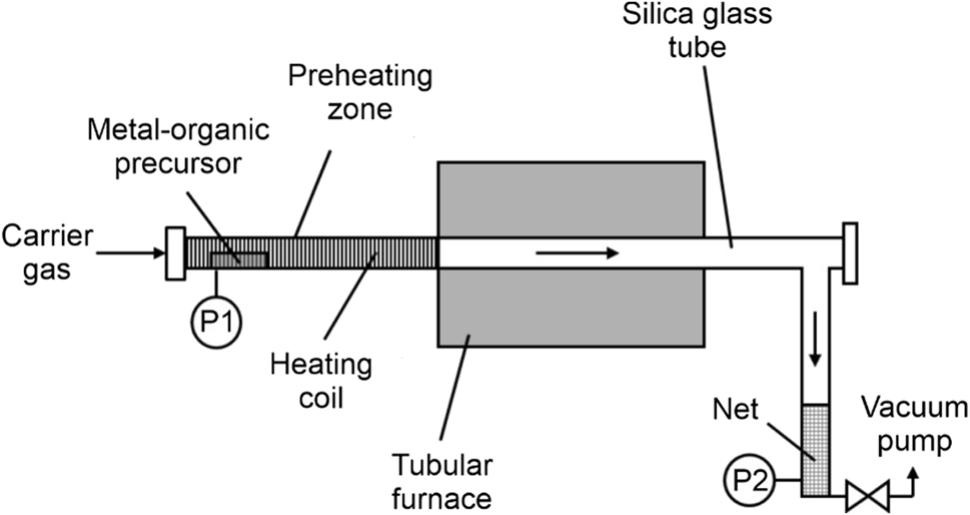
Flow reactor system used to produce ZnO and TiO_2_ nanoparticles via metal–organic chemical vapor synthesis

We used metal–organic chemical vapor synthesis (MO-CVS) to produce TiO_2_ and ZnO nanoparticle powders. Details of the synthesis route are provided in the references [43, 44]. The approach is based on the decomposition of a metal–organic precursor in a flow reactor system (Fig. 6) at elevated temperatures and pressures of *p* = 15 or 30 mbar for ZnO and TiO_2_ synthesis, respectively. A heating coil guarantees the evaporation of the metal–organic precursor, which is then carried by gas transport (O_2_ or Ar) into the reaction zone. A tubular furnace ensures the decomposition of the evaporated metal–organic precursor inside the reaction zone. There, homogeneous nucleation leads to nanoparticle formation in the gas phase. Low synthesis pressures and short residence times in the reaction zone ensure the production of small particles with narrow size distributions in the *d* < 20 nm range. Respective temperatures (*T*_preheating_ and *T*_furnace_), adjusted carrier gas flow rates (Ar, O_2_), and the synthesis pressure (*p*) for both metal oxide systems (Table 2) are kept constant during the entire process of particle collection.

**Table 2:** Synthesis parameters of CVS – ZnO and TiO_2_ nanoparticles

	ZnO	TiO_2_
Precursor	Zinc acetate dihydrate Zn(CH_3_COO)_2_ ∙ H_2_O Sigma Aldrich (99%)	Titanium (IV) isopropoxide Ti[OCH(CH_3_)_2_]_4_ Sigma Aldrich (99.99%)
*T*_preheating zone_/K	548	383
*T*_furnace_/K	1073	1073
Carrier gas	Oxygen	Argon
Gas flow/sccm	640	840
Pressure/mbar	15	30

After production, the TiO_2_ and ZnO nanoparticle powders are transferred into fused silica cells, which allow alternating thermal activation cycles of the powder in high vacuum (*p* < 10^–5^ mbar) and in an O_2_ atmosphere. Stepwise heating to 673 and 873 K for ZnO and TiO_2_, respectively, at high vacuum (*p* < 10^–5^ mbar) eliminates surface contaminations from the particles. After reaching the final temperature, we expose the powder to molecular oxygen for 30 min and then evacuate for 15 min (*p* < 10^–5^ mbar). Oxygen addition and subsequent evacuation are performed two times to induce the decomposition of carbon remnants from the metal–organic precursor and convert them into volatile CO and CO_2_, which are removed by continuous pumping. Fresh oxygen is added during cooling the TiO_2_ nanoparticle powder to room temperature to prevent oxygen vacancy formation.

### Electrospinning

The nanoparticle powders were dispersed in polyvinyl alcohol (PVA) (98.0–98.8% hydrolyzed M.W. 31,000–50,000) polymer solutions as a precursor for electrospinning. First, we dissolved a defined amount of polymer powder in high-purity water (23 g/100 cm^3^) under continuous stirring at 90 °C for 3 h, adjusting the concentration to 23% w/v. Next, we added a defined amount of nanoparticle powder (60 mg/cm^3^) to the polymer solutions under vigorous stirring. The stirring was kept for 30 min prior to 15 min of ultrasonic radiation treatment of the dispersion. We used electrospinning to generate continuous fibers with a diameter in the micrometer and nanometer range. For the materials described here, we applied an 18 kV potential, used a pumping rate of 0.05 cm^3^/h, and kept a 20 cm distance between the tip of the needle and the collector. Neglecting the increase of the dispersions volume after dispersing the powder in the polymer solution, we estimate a production capacity, i.e., a deposition rate of 3 mg metal oxide nanoparticles encapsulated inside fibers per hour.

### Thermal treatment

We annealed the electrospun nanoparticle–polymer fibers to remove the polymer matrix and to produce nanoparticle-based fibers. For this purpose, the fibers were spun directly on gold TEM grids and placed in a horizontal high-temperature ceramic tube furnace (Nabertherm RHTH80-300/16). This furnace guarantees stable final temperatures (± 5 K) and allows us to perform annealing in defined gas flows. A continuous flow of synthetic air (40 cm^3^/min Ar and 10 cm^3^/min O_2_) was kept during the entire sintering protocol. Specimens were heated at 5 K min^−1^ to the final temperature of 1023 K and dwelled at this temperature for 24 h before cooling to room temperature. Such treatment is efficient to remove the entire polymer, as can be stated from the thermogravimetric analysis (TGA) results that show no further mass loss/decomposition above 1023 K.

### Structure characterization

Electron microscopy images were acquired using scanning electron microscopy (SEM) and transmission electron microscopy (TEM). The SEM instrument (Zeiss FE-Ultra Plus 55) is equipped with a field-emission gun and Gemini lenses and was used at short working distances of around 3 mm and an accelerating voltage between 5 and 10 kV, with InLens and SE detectors. The diameters of the electrospun fibers were evaluated from the SEM images by measuring the diameter with the software program ImageJ (V1.52a). The TEM (JEOL JEM-F200 TEM) was operated at 200 ​kV and is equipped with a cold field-emission electron source and a TVIPS F216 2 k by 2 k CMOS TEM camera. Particle size distribution plots before and after sintering and fiber diameters after thermal treatment were obtained from TEM images using the EM Measure software program from TVIPS.
